# Computerized cyclic voltammetric detection after HPLC of the antineoplastic agents etoposide, teniposide, adriamycin and its metabolite adriamycinol in urine samples

**DOI:** 10.1155/S1463924689000246

**Published:** 1989

**Authors:** H. H. J. L. Ploegmakers, P. A. Moritz, P. J. M. M. Toll, W. J. van Oort

**Affiliations:** 1 Department of Analytical Pharmacy State University of Utrecht Catharijnesingel 60 Utrecht 3511 GH The Netherlands; 2 Groot Ziekengasthuis Hospital Nieuwstraat 34 's-Hertogenbosch 5211 NL The Netherlands

## Abstract

A computerized electrochemical detection system for application after HPLC, provided with a cyclic voltammetric oxidative and reductive module, is described for the on-line qualitative determination of electroactive antineoplastic agents and metabolites in urine samples, collected from cancer patients, following intravenous administration.

The application of two cyclic voltammetric detection modes provides an insight into both oxidative and reductive electrode reactions of compounds, passing the detector and the occurrence of (it)reversible chemical and electrochemical processes at the electrode surface. In this way, redox properties of drugs and metabolites characteristic of their molecular structure, can be established, which may provide information related to their (enzymatic) bioactivation.

In the cyclic voltammetric mode, the system permits automatic detection of a compound in the cell, recording, storage and plotting of voltammograms and calculation of the retention times, the half-wave potentials and the peak potentials of each scan of all individual compounds. For routine use, storage of 68 voltammograms on-line is sufficient for the analysis of biological samples in clinical-pharmacological research. Special attention has been paid to automatic, multi-reference-point component detection.

Based on their concentrations in urine, the oxidative cyclic voltammetric mode, using a glassy carbon electrode, permits the determination of etoposide and teniposide, whereas the reductive cyclic voltammetric mode, with a static mercury drop electrode, permits the determination of adriamycin and its metabolite adriamycinol.


Journal of Automatic Chemistry Vol. 11, No. 3 (May–June 1989), pp. 106–112

### Computerized cyclic voltammetric detection after HPLC of the antineoplastic agents etoposide, teniposide, adriamycin and its metabolite adriamycinol in urine samples

**H. H. J. L. Ploegmakers and P. A. Moritz**
*Department of Analytical Pharmacy, State University of Utrecht, Catharijnesingel 60, 3511 GH Utrecht, The Netherlands*
**P. J. M. M. Toll**
*Groot Ziekengasthuis Hospital, Nieuwstraat 34, 5211 NL's-Hertogenbosch, The Netherlands*
**and W. J. van Oort**
*Department of Analytical Pharmacy, State University of Utrecht, Catharijnesingel 60, 3511 GH Utrecht, The Netherlands*

*A computerized electrochemical detection system for application after HPLC, provided with a cyclic voltammetric oxidative and reductive module, is described for the on-line qualitative determination of electroactive antineoplastic agents and metabolites in urine samples, collected from cancer patients, following intravenous administration.*

*The application of two cyclic voltammetric detection modes provides an insight into both oxidative and reductive electrode reactions of compounds, passing the detector and the occurrence of (ir)reversible chemical and electrochemical processes at the electrode surface. In this way, redox properties of drugs and metabolites characteristic of their molecular structure, can be established, which may provide information related to their (enzymatic) bioactivation.*

*In the cyclic voltammetric mode, the system permits automatic detection of a compound in the cell, recording, storage and plotting of voltammograms and calculation of the retention times, the half-wave potentials and the peak potentials of each scan of all individual compounds. For routine use, storage of 68 voltammograms on-line is sufficient for the analysis of biological samples in clinical–pharmacological research. Special attention has been paid to automatic, multi-reference-point component detection.*

*Based on their concentrations in urine, the oxidative cyclic voltammetric mode, using a glassy carbon electrode, permits the determination of etoposide and teniposide, whereas the reductive cyclic voltammetric mode, with a static mercury drop electrode, permits the determination of adriamycin and its metabolite adriamycinol.*

### Introduction

For on-line detection after high-performance liquid chromatography (HPLC), many methods of analysis are available, based on various physicochemical principles such as UV and fluorescence spectrometry and amperometry. In general, all these modes of detection are able to provide quantitative information for suitable compounds. Selectivity can be improved both by optimizing the chromatographic system and by selecting the optimum wavelength(s) or potential of the detection system used. Both parameters are determined by the molecular structure of the compound(s) to be analysed and by the kind and amounts of interfering compounds in the sample.

When qualitative information is needed, more sophisticated methods are required such as scanning UV spectrometry or cyclic voltammetry. Such systems can provide considerable amounts of qualitative information about the compounds passing the detector. In practice, large information streams resulting from such complex measuring techniques mostly require the application of a computer.

In a previous paper [1], we described such a computerized cyclic voltammetric detection system in HPLC. Owing to the large background current, which is inherent in cyclic voltammetry using a glassy carbon electrode, the detection limit for quantitative analysis proved to be higher than that of voltammetric detection systems, applied at constant potential. Nevertheless, that investigation showed that significantly more qualitative information about compounds can be achieved by cyclic voltammetric detection than voltammetric constant potential detection.

The relatively high detection limit of such a computerized cyclic voltammetric system has hampered the qualitative analysis of low-dosage drugs and metabolites in plasma and serum up to now [1, 2]. However, the volume of urine samples is rarely limited. The analysis of parent drugs and possible metabolite(s) in urine, a major elimination route of drugs, may yield information on the kinetics and metabolism.

To show the performance of the technique, we selected the anticancer drugs etoposide, teniposide and adriamycin as model compounds. In biological samples, e.g., urine from patients following intravenous administration, such compounds are always accompanied by several interfering oxidizable and reducible endogenous compounds. These cytostatic compounds are also believed to possess an extended metabolism pattern, creating the need for increased qualitative information, applying relatively simple technology if possible.

As most of these drugs need enzymatic oxidative or reductive bioactivation, producing, e.g., relatively stable free radicals, fast-scanning electrochemical detection modes may provide other or additional information about

0142–0453/89 $3.00 © 1989 Taylor & Francis Ltd.

the reaction mechanisms compared with scanning UV spectrometry.

Etoposide (VP 16-213) and teniposide (VM 26) are epipodophyllotoxin derivatives (*Podophyllum peltatum* L. and *Podophyllum emodi* Wallich), active against several types of tumours, particularly small cell carcinoma of the lung, adenocarcinoma, ovarian cancer and squamous cell cancer.

Etoposide and teniposide are known to be excreted in the urine in the form of the unchanged parent drug and the hydroxy acid derivative metabolite [3]. Both compounds differ significantly in concentration, polarity and lipophilicity.

For the determination of etoposide and teniposide, several HPLC systems have been reported, mostly using UV [4–8] and fluorescence detection [9, 10]. For the determination of the hydroxy acid metabolite, a method involving fluorescence detection was described by Strife *et al.* [4].

As etoposide and teniposide possess a phenolic hydroxy group, oxidative electrochemical detection is also applicable [11–14]. A routinely applicable method for the determination of the hydroxy acid metabolites of both drugs using amperometric detection has been described by Sinkule and Evans [12]. Holthuis [3] concluded that the quantitative HPLC of etoposide, using oxidative amperometry, provides more sensitive detection (absolute detection limit 250 pg) than UV (detection limit 100–500 ng/ml in plasma) and fluorescence methods (detection limit 50 ng/ml in plasma).

Adriamycin, an anthracycline antibiotic produced by *Streptomyces* species, is active and frequently used against leukaemias and mammary carcinomas. It is excreted in the urine in the form of the unchanged drug and its metabolite adriamycinol, the 13-hydroxy derivative of adriamycin.

Several HPLC systems for the analysis of anthracyclines have been published using UV [15–19] and fluorescence detection [20–26]. Anthracyclines possess both phenolic hydroxy groups, permitting oxidative electrochemical detection [27, 28], and a *p*-quinoid system, permitting reductive electrochemical detection. Application of the latter method avoids interference from catecholamines during detection.

So far, no investigations on the analysis of these compounds using HPLC with electrochemical detection, exploiting its reductive properties, appear to have been published; this may offer promising opportunities for qualitative analysis. This investigation, using relatively well known drugs and metabolites as model compounds, was aimed at demonstrating the applicability and reliability of computerized scanning electrochemical detection, in the oxidative and reductive mode, with real biological samples collected from cancer patients.

### Experimental

### *Hardware*

The heart of the on-line pilot voltage-generating and data-acquisition system used is an Apple IIe computer (figure 1), equipped with 64 kbytes of on-board RAM and two 125K Apple floppy disk drives. We combined this computer with an Axlon 320K Ramdisk, a 12-bit, 16-channel Digilog A/D converter, a 12-bit D/A converter (made by coupling a California Computer Systems 7720 parallel interface and an Analog Devices AD DAC80 D/A converter), a California Computer Systems Model 7424 real-time clock, an Itoh 8510 parallel printer with screen-dump facility (Kronemuis APL 13 graphic printer interface) and a Hewlett-Packard 7470A serial plotter.

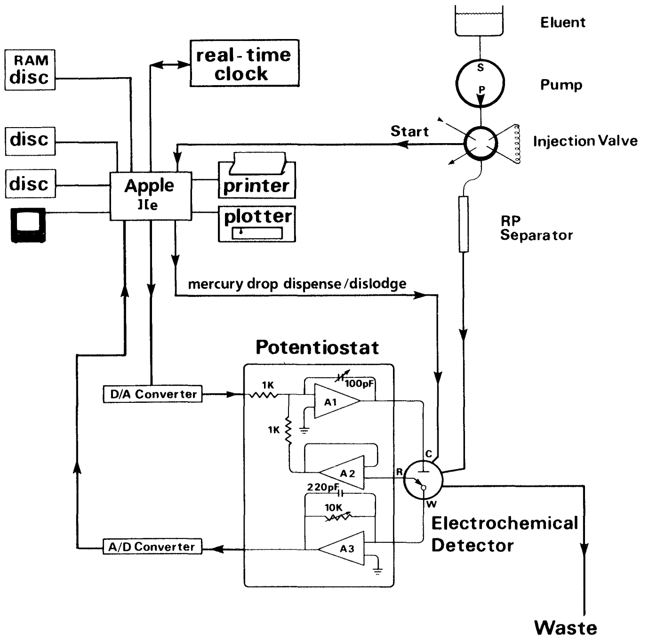

*Figure 1. Schematic diagram of the chromatographic, electrochemical and computer system.*

To this computer system, a Metrohm 641 VA-Detector potentiostat (output 1 V full-scale), a Princeton Applied Research 310 polarographic detector for reductive detection or a laboratory made, centrally injected, electrochemical cell for oxidative detection are connected.

The laboratory-made cell consists of a mini glassy carbon electrode (GCE) (Metrohm EA-286/1, diameter 3 mm), placed in the centre of a stainless-steel ring auxiliary electrode. The distance between the GCE and the jet can be adjusted by a micrometer on which the electrode is mounted. The eluent stream is directed perpendicularly to the surface of the GCE. A laboratory-made Ag/AgCl reference electrode with a Vycor glass-tipped jacket is placed close to the working and auxiliary electrodes. The diameter of the outlet jet of the eluent of the chromatographic system is 0·11 mm. The cell is submerged in a vessel containing the mobile phase.

*Table 1. Functions of the programs.*

| Language | Program name | Function |
|---|---|---|
| Basic | HPLC | HPLC with electrochemical detection at constant potential |
| Assembly | CLOCK | To reset and restart real-time clock and data storage in HPLC/ML |
| Assembly | HPLC/ML | Measuring, data storage, timing and drop dislodging |
| Basic | PARAMETER | Parameters for cyclic voltammetry |
| Assembly | DUMMY | To stabilize SMDE or GCE |
| Assembly | THRESHOLD | Detection of the passage of a compound through the detector |
| Assembly | DETERMINATION | To store a cyclic voltammogram of a compound |
| Basic | CALCULATION | Display of voltammograms on screen and production of a paper copy; to filter data and to calculate peak potential and 'half-wave potential' |

The chromatographic system consists of a Model 510 solvent delivery system and a Model U6K septumless injector (both from Waters Associates). The chromatographic columns and mobile phases used depended on the compounds being investigated.

*Software and interfacing*

To control the mercury drop knocker and the nitrogen stream in the polarographic detector by the computer, interfacing was achieved by connecting annunciator outputs of the Apple computer to the rear side connector J1 of the Princeton Applied Research 310 polarographic detector.

In order to detect the moment of injection, the switch in the chromatographic injector was connected to an input on the game-connector of the Apple computer by means of a 74LS00 flip-flop: if the content of address $C061 (hexadecimal) changes from $FF (hexadecimal) to $7F (hexadecimal), this indicates that an injection has been made.

As the potentiostat attenuates the pilot voltage ten times, the D/A converter signal has to be amplified (gain: +10 and −10), enabling the desired potential of the glassy carbon and mercury working electrodes to be set. To adapt the input signal to the voltage range of the A/D converter, the $i/E$ converter of the potentiostat was connected to the A/D converter by an amplifier/attenuator/offset circuit.

HPLC (Table 1) is a program for performing HPLC with oxidative or reductive electrochemical detection at constant potential at the GCE and the static mercury electrode. It deaerates the mobile phase in the detector cell for 10 min, investigates the number of components present in the sample and establishes the retention times of these substances.

On switching on the detector cell, the current is usually out of range (overload) owing to stabilizing processes at the electrode surface. Therefore, the CLOCK program, a subroutine of the HPLC/ML program, permits resetting and restarting of the real-time clock, the data storage and the high-resolution graphics screen after a stabilizing period without losing the variables.

Applying the PAR 310 detector each time a new drop is dispensed, the volume of the drop reaches its final volume very fast and, shortly afterwards, the capacity current reaches its minimum value. Therefore, by inserting a software delay of 1 s after dispensing the mercury drop, the current can reach a constant value, almost freed from capacity current. After this delay, 16 current measurements are executed and averaged, using a machine language routine. The result is stored in the memory and plotted on screen in the high-resolution graphics mode.

The continuous flow of the eluent against the fragile mercury drop sometimes blows the drop off the capillary, resulting into a sudden current cut-off. The frequency of this process proved to be influenced by the flow-rate, the composition of the mobile phase and the potential of the working electrode.

The computer can be used to compensate for this event. In the HPLC program, all stored current values are compared with the preceding current measurement; if the difference is less than a user-defined value, then a current cut-off is detected and this current value is discarded and replaced by its predecessor; in this way, the curve is partially smoothed.

The HPLC program also allows the saving of data in addition to parameters on disk, smoothing the chromatogram, using a machine language 11-point parabolic filter routine, plotting the curves, calculating the retention times and plotting this information in combination with the peaks.

In a previous paper [1], a cyclic voltammetric detection system after HPLC was described in which a GCE was used. The on-line cyclic voltammograms of etoposide and teniposide obtained in this way show waves. To detect a compound in the electrochemical cell in this system, it is sufficient to compare the current at a certain potential (e.g., +700 mV) with the corresponding current measurement of the blank scan; if the current value is higher then the corresponding blank value, increased with a user-defined threshold, this indicates a compound passing the detector.

The on-line cyclic voltammograms of adriamycin and adriamycinol obtained using the static mercury drop electrode show peaks, however. In this event, the current decreases at the end of the scan. Therefore, a current comparison at a single potential value at the end of each scan is not sufficient. After stabilizing the system, using six dummy scans (DUMMY program), the THRESHOLD program is executed. After storage of a blank scan, each hundredth current measurement is increased with a user-defined threshold value and stored in the memory. On scanning, each 100th current measurement is compared with the corresponding stored blank current value. If a current measurement is larger, this indicates that a compound is passing the detector and then a detection flag is set. After completion of the scan, the computer checks the flag and, if set, the program jumps to the storage DETERMINATION subroutine. This subroutine stores the scan, accompanied with real time, in the Ramdisk. After completing this subroutine, the program returns to the THRESHOLD scanning and comparison mode.

As the voltammograms of the parent drugs and decomposition products show both waves and peaks, the CALCULATION program is adapted with a half-wave potential and a peak potential calculation routine. When applying the CALCULATION half-wave potential or peak potential calculation subroutine, it is necessary to smooth the curve first. In a previous study [1], a parabolic filter method, written in Applesoft Basic, was applied. The software of this method can easily be programmed but it is time consuming. Because of the favourable data handling obtained in this way, a 6502 machine language 11-point parabolic filter program was written. This program filters the data very quickly in the calculations.

*Etoposide*

Etoposide (VP 16-213) was kindly supplied by Bristol-Myers Nederland (Bussum, The Netherlands). The mobile phase consists of methanol – 0·020 M phosphate buffer (pH 7) (45 : 55, w/w); the samples are injected on to a LiChrosorb RP-18 (10 μm) analytical column (30 cm × 3·9 mm i.d.). As a reference solution, etoposide is dissolved in methanol (analytical-reagent grade, Merck) at a concentration of 4·796 mg in 10 ml. Under these conditions, the retention time of etoposide is 7 min 26 s.

*Sample treatment*

To determine etoposide in human urine, 25 ml of urine, after mixing with 2·5 ml of 0·1 M phosphate buffer (pH 7·3), are extracted twice with 25 ml of 1,2-dichloroethane (DCE) [13]. The organic layer is removed and evaporated under a stream of nitrogen at 30 °C. The residue is dissolved by sonication in 1·00 ml of methanol. For determinations in the cyclic voltammetric mode, an aliquot of 10 μl is injected.

*Teniposide*

Teniposide (VM-26) was kindly supplied by Bristol-Myers Nederland. The mobile phase consists of methanol – 0·02 M phosphate buffer (pH 7) (55 : 45 w/w); the samples are injected on to the same column as for etoposide. As a reference solution, teniposide is dissolved in methanol at a concentration of 5·852 mg in 10 ml. Under these conditions the retention time of teniposide is 7 min 8 s.

*Sample treatment*

To determine teniposide in human urine, 50 ml of urine, after mixing with 5 ml of 0·1 M phosphate buffer (pH 7·3), are extracted twice with 50 ml of DCE [3]. The organic layer is removed and evaporated under nitrogen at 30 °C. The residue is dissolved by sonication in 1·00 ml of methanol. For determinations in the cyclic voltammetric mode, an aliquot of 25 μl is injected.

*Adriamycin and adriamycinol*

Adriamycin hydrochloride and adriamycinol were kindly supplied by Carlo Erba (Milan, Italy). The mobile phase consists of methanol – 0·02 M phosphate buffer (pH 4·2) (40 : 60 w/w); the samples are injected on to a LiChrosorb RP-8 (50432 $C_8$) (5 μm) reversed-phase column (12·5 cm × 4 mm i.d.). As references, methanolic solutions of adriamycin (0·497 mg/ml) and adriamycinol (0·438 mg/ml) are used. Under these conditions, the retention times of adriamycinol and adriamycin are 6 min 4 s and 7 min 32 s, respectively.

*Sample treatment*

Several methods for the extraction of adriamycin and its main metabolite adriamycinol have been described. Some of these methods include liquid–liquid extraction [20–24] and adsorption chromatography using a Celite column [25], a loop column [27] or a syringe cartridge [26]. As the concentrations of the drug and its metabolite in urine are low in relation to the sensitivity of cyclic voltammetric detection, it is necessary to extract a relatively large volume of urine.

To concentrate adriamycin and adriamycinol, 60 ml of human urine [buffered by 5 ml 0·1 M phosphate buffer (pH 8·0)] are extracted twice with 100 ml of chloroform–methanol (9 : 1) [23]. After evaporating the organic phase under a stream of nitrogen at 30 °C, the residue is dissolved in 150 μl of methanol. For determination in the cyclic voltammetric mode, 100 μl of the methanolic solution are injected.

### Results and discussion

*Etoposide*

In on-line experiments, the anodic (forward) scan of the cyclic voltammogram of etoposide, present in the urine extract, shows two waves (figure 2). This behaviour is similar to that of the pure compound [1]. The 'half-wave potentials' of these waves are +383 mV ($n$ = 5, SD = 5 mV) and +722 mV ($n$ = 5, SD = 3 mV). As the limiting

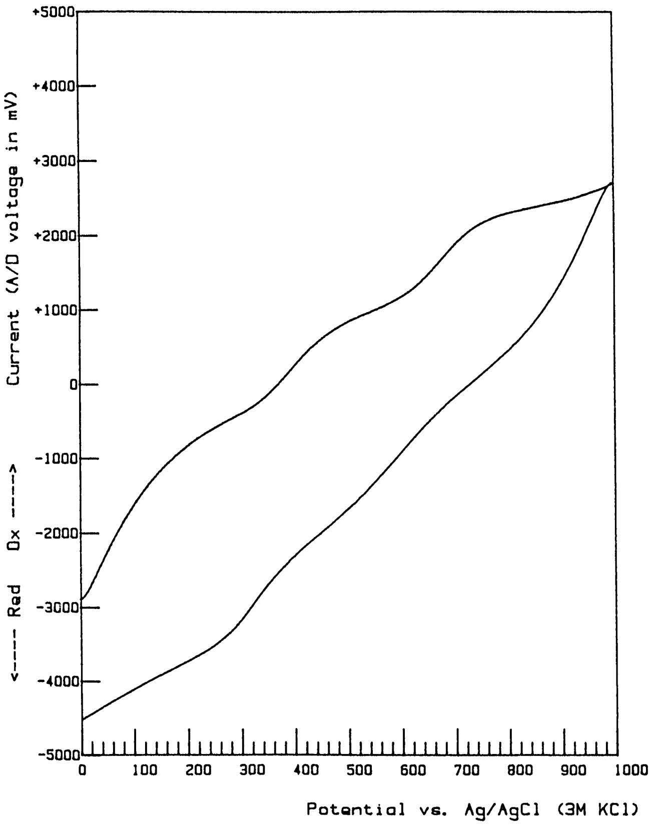

*Figure 2. On-line cyclic voltammogram of etoposide (VP 16-213) in a urine extract after HPLC separation. Conditions: 10 μl injected; current range, 0·5 μA full-scale; flow-rate, 1 ml/min; scan speed, 300 mV/s; mobile phase, methanol – 0·020* M *phosphate buffer (pH 7) (45 : 55 w/w); column, LiChrosorb RP-18.*

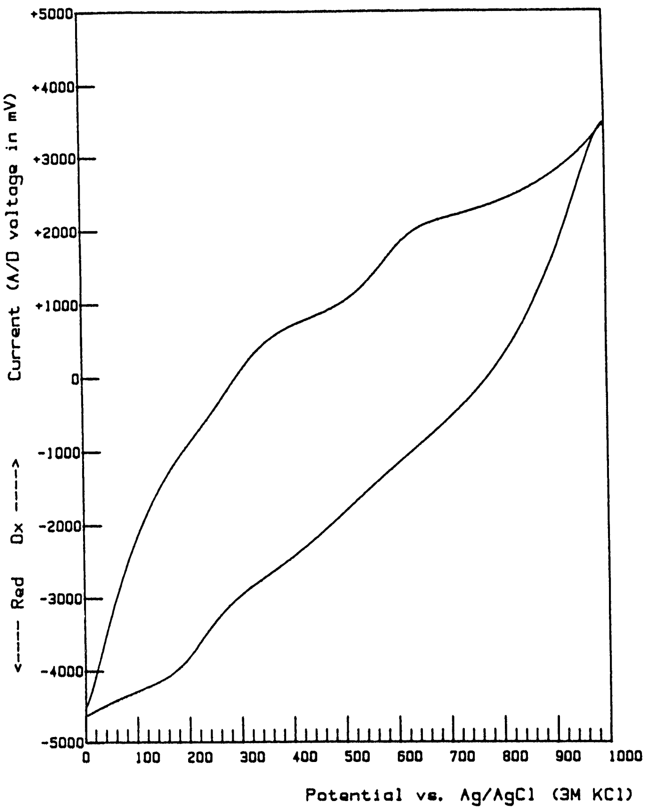

*Figure 3. On-line cyclic voltammogram of teniposide (VM-26) in a urine extract after HPLC separation. Conditions: 25 μl injected; current range, 0·5 μA full-scale; flow-rate, 1 ml/min; scan speed, 300 mV/s; mobile phase, methanol – 0·020* M *phosphate buffer (pH 7) (55 : 45 w/w); column, LiChrosorb RP-18.*

current in flow systems is expected to be governed both by diffusion and by convection, the terms '$E_{1/2}$' or 'half-wave potential', in quotation marks, have been used, to be distinguished from the conventional half-wave potential, $E_{1/2}$, known from classical d.c. polarography [29].

*Teniposide*

In on-line experiments, the anodic (forward) scan of the cyclic voltammogram of teniposide, present in the urine extract, shows two waves (figure 3). This behaviour is similar to that of the pure compound [1]. The 'half-wave potentials' are +300 mV ($n$ = 5, SD = 2 mV) and +584 mV ($n$ = 5, SD = 12 mV).

*Adriamycin and adriamycinol*

For the HPLC of adriamycin, several workers have used an acetonitrile-containing mobile phase [20, 21, 25–27]. In the electrochemical system described, acetonitrile proved to be unsuitable; in the reductive electrochemical detection mode, trace levels of oxygen, present in the sample, show a chromatographic behaviour similar to that of adriamycin. A methanol – 0·02 M phosphate buffer (pH 4·2) (40 : 60 w/w) mobile phase and a LiChrosorb RP-8 column proved to solve this problem. This mobile phase permits the separation of adriamycin and adriamycinol and shows sufficient retention with respect to oxygen.

In batchwise experiments using the static mercury drop electrode (SMDE), adriamycin and adriamycinol show an adsorption 'pre-peak' and a reduction peak in the cathodic (forward) scan and one oxidation peak in the anodic (backward) scan. The peak potentials in the cathodic and anodic scans of adriamycin and adriamycinol are −588 and −440 mV ($n$ = 4, SD = 2 mV) and −547 and −440 mV ($n$ = 3, SD = 3 mV), respectively.

As the peak potentials of the two compounds differ only slightly, separation techniques are needed for differentiation, in both stock solutions and biological samples.

In HPLC with cyclic voltammetric detection using the same electrodes and the same scan speed, both compounds show one reduction peak in the forward scan and one oxidation peak in the backward scan; the peak potentials of the cathodic and anodic scans of adriamycin and adriamycinol are −583 and −440 mV ($n$ = 5, SD = 3 mV) and −538 and −440 mV ($n$ = 3, SD = 3 mV), respectively.

Analysis of the urine extract demonstrate that both adriamycin and adriamycinol are detectable in the reductive cyclic voltammetric mode. Adriamycin shows one reduction peak (figure 4a), but probably the concentration of adriamycinol in the urine is too low to form a well defined peak in the cathodic scan (figure 4b).

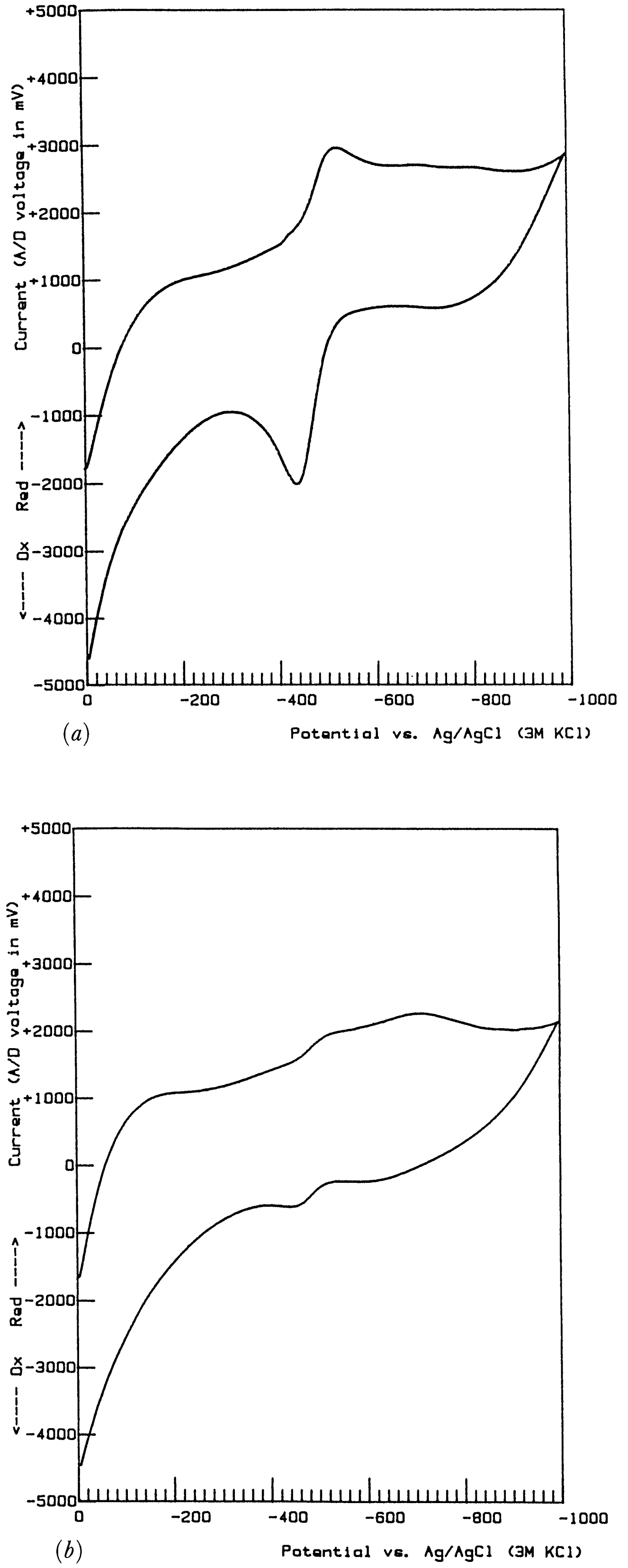

*Figure 4. On-line reductive cyclic voltammogram of (a) adriamycin and (b) adriamycinol in a urine extract after HPLC separation Conditions: 100 μl injected; current range, 50 nA full-scale; flow-rate, 2 ml/min; scan speed, 300 mV/s; mobile phase, methanol – 0·02* M *phosphate buffer (pH 4·2) (40 : 60 w/w); column, LiChrosorb RP-8.*

The peak potentials of the forward and backward scans of adriamycin are −529 and −438 mV ($n = 5$, SD = 4 mV), respectively, whereas the peak potential in the forward scan of adriamycinol is −520 mV.

The on-line experiments described above show substantial differences in the observed cathodic peak potentials of adriamycin (−583 and −529 mV) when analysing the pure compound and a urine extract at different concentrations. In the anodic scans, however, the peak potentials are the same and the peak heights are almost identical. Probably this phenomenon is caused by adsorption. Analyses for adriamycin and adriamycinol are always hampered (or even supported) by strong adsorption, e.g., on a glass surface [21], mercury electrodes [30] or carbon paste electrodes [31]. The theory and practical applications of the influence of adsorption on cyclic voltammetric curves have been described by Wopschall and Shain [32–34]. However, the adsorption of adriamycin is complex and the lack of correlation between the theory and the experimental results is not yet understood. At concentrations where adsorption itself becomes important, only qualitative comparisons can be made with theory [33], but a semi-empirical method has been developed which appears to be generally applicable [34]. However, the described method permits the application of extra parameters to investigate adriamycin compared with batch-wise voltammetry, such as flowing systems and time dependence. The adsorption behaviour might be of importance in understanding its behaviour *in vivo*.

### Conclusions

For qualitative analysis in particular, the computerized cyclic voltammetric detection system after HPLC described here has several promising properties compared with previously described amperometric systems at constant potential [11–14]. The ultimate sensitivity for the analysis of biological samples has not yet been reached and needs to be optimized. As the system is provided with an oxidative and reductive module, all kinds of electroactive compounds can be investigated.

The cyclic voltammetric system described has been applied to the identification of etoposide and teniposide in urine samples using the oxidative mode and adriamycin and adriamycinol using the reductive mode.

In a previous study [1] we calculated the 'half-wave potentials' of etoposide and teniposide, using the same electrodes in the same system. However, the 'half-wave potentials' of these two compounds calculated here have more positive values. Probably this difference is caused by the decreased reactivity of the (same) GCE after 2 years of intensive use. As glassy carbon is known not to be a homogeneous and defined material like mercury, the application of a new electrode will show a different reactivity. Therefore, batch-wise and on-line cyclic voltammetry need to be performed at the same time with the same GCE for optimum comparisons. In the reductive cyclic voltammetric mode, the calculated peak potentials in the cathodic scan for adriamycin present in a stock solution and a urine extract differ by 54 mV, whereas the peak potentials in the anodic scan are identical; the anodic peaks are higher than the cathodic peaks. These results are probably caused by adsorption effects, which differ in flowing and unstirred solutions.

The model compounds were selected to show that the described methods could substantially yield more information than existing methods. In the future, special attention will be paid to sensitivity. Methods are being developed, based on the method described here, for investigating the relationship between pH and the electrochemical properties of anticancer drugs both *in vitro* and *in vivo*.

### References

1. Ploegmakers, H. H. J. L., Mertens, M. J. M., and van Oort, W. J., *Analytica Chimica Acta*, **174** (1985), 71.
2. Ploegmakers, H. H. J. L., Mertens, M. J. M., and van Oort, W. J., *Journal of Automatic Chemistry*, **9** (1987), 149.
3. Holthuis, J. J. M., *Thesis*, State University of Utrecht (1985).
4. Strife, R. J., Jardine, I., and Colvin, M., *Journal of Chromatography*, **182** (1980), 211.
5. Farina, P., Marzillo, G., and D'Incalci, M., *Journal of Chromatography*, **222** (1981), 141.
6. Allen, L. M., *Journal of Pharmaceutical Science*, **69** (1980), 1440.
7. Scalzo, A. J., Comis, R., Fitzpatrick, A., Issell, B. F., Nardella, P. A., Pfeffer, M., Sneyth, R. D., and Harker, D. R., *Proceedings of the American Society for Clinical Oncology*, **18** (1982), 128.
8. Holthuis, J. J. M., van Oort, W. J., and Pinedo, H. M., *Analytica Chimica Acta*, **130** (1981), 23.
9. Strife, R. J., Jardine, I., and Colvin, M., *Journal of Chromatography*, **224** (1981), 168.
10. Werkhoven-Goewie, C. E., Brinkman, U. A. Th., Frei, R. W., de Ruiter, C., and de Vries, J., *Journal of Chromatography*, **276** (1983), 349.
11. Evans, W. E., Sinkule, J. A., Crom, W. R., Dow, L., Look, A. T., and Rivera, G., *Cancer Chemotherapy and Pharmacology*, **7** (1982), 147.
12. Sinkule, J. A., and Evans, W. E., *Journal of Pharmaceutical Science*, **73** (1984), 164.
13. Holthuis, J. J. M., Romkens, F. M. G. M., Pinedo, H. M., and van Oort, W. J., *Journal of Pharmaceutical and Biomedical Analysis*, **1** (1983), 89.
14. Rideout, J. M., Ayres, D. C., Lim, C. K., and Peters, T. J., *Journal of Pharmaceutical and Biomedical Analysis*, **2** (1984), 125.
15. Benvenuto, J. A., Anderson, R. W., Kerkhof, K., Smith, R. G., and Loo, T. L., *American Journal of Hospital Pharmacy*, **38** (1981), 1914.
16. Haneke, A. C., Crawford, J., and Aszalos, A., *Journal of Pharmaceutical Science*, **70** (1981), 1112.
17. Poochikian, G. K., Cradock, J. C., and Flora, K. P., *American Journal of Hospital Pharmacy*, **38** (1981), 483.
18. Karlsen, J., Thonnesen, H. H., Olsen, I. R., Sollien, A. H., and Skobba, T. J., *Nordica Pharmaceutica Acta*, **45** (1983), 61.
19. Thomas, A. H., Quinlan, G. J., and Gutteridge, J. M. C., *Journal of Chromatography*, **299** (1984), 489.
20. Pierce, R. N., and Jatlow, P. I., *Journal of Chromatography*, **164** (1979), 471.
21. Bots, A. M. B., van Oort, W. J., Noordhoek, J., van Dijk, A., Klein, S. W., and van Hoesel, Q. G. C. M., *Journal of Chromatography*, **272** (1983), 421.
22. Cummings, J., *Journal of Chromatography*, **341** (1985), 401.
23. Peters, J. H., and Murray, J. F., Jr., *Journal of Liquid Chromatography*, **2** (1979), 45.
24. Shinozawa, S., Mimaki, Y., Araki, Y., and Oda, T., *Journal of Chromatography*, **196** (1980), 463.
25. Eksborg, S., Ehrsson, H., and Andersson, I., *Journal of Chromatography*, **164** (1979), 479.
26. Kotake, A. N., Vogelzang, N. J., Larson, R. A., and Choporis, N., *Journal of Chromatography*, **337** (1985), 194.
27. Akpofure, C., Riley, C. A., Sinkule, J. A., and Evans, W. E., *Journal of Chromatography*, **232** (1982), 377.
28. Schwartz, H. S., and Paul, B., *Cancer Research*, **44** (1984), 2480.
29. Bond, A. M., in *Modern Polarographic Methods in Analytical Chemistry* (Marcel Dekker, New York, 1980), p. 227.
30. Kano, K., Konse, T., Nishimura, N., and Kubota, T., *Bulletin of the Chemical Society of Japan*, **57** (1984), 2383.
31. Chaney, E. N., Jr., and Baldwin, R. P., *Analytical Chemistry*, **54** (1982), 2556.
32. Wopschall, R. H., and Shain, I., *Analytical Chemistry*, **39** (1967), 1514.
33. Wopschall, R. H., and Shain, I., *Analytical Chemistry*, **39** (1967), 1527.
34. Wopschall, R. H., and Shain, I., *Analytical Chemistry*, **39** (1967), 1535.

